# Diagnostic performance of culture filtered protein 10‐specific perforin in pediatric patients with active tuberculosis

**DOI:** 10.1002/jcla.23477

**Published:** 2020-07-16

**Authors:** Qinzhen Cai, Xin Shen, Hongze Li, Cong Yao, Na Sun, Jun Wang, Huan Wu, Chunhui Yuan, Jie Xiang, Yun Xiang

**Affiliations:** ^1^ Department of Laboratory Medicine Wuhan Children's Hospital Tongji Medical College Huazhong University of Science and Technology Wuhan China; ^2^ Department of Laboratory Medicine Hubei University of Chinese Medicine Wuhan China; ^3^ Department of Laboratory Medicine Wuhan Jinyintan Hospital Wuhan China; ^4^ Health Care Department Tongji Medical College Wuhan Children's Hospital Huazhong University of Science and Technology Wuhan China

**Keywords:** CFP10, ESAT6, pediatric patients, perforin, tuberculosis diagnosis

## Abstract

**Background:**

*Mycobacterium tuberculosis* (Mtb)‐specific perforin were significantly increased in patients with tuberculosis. This study aims to evaluate the diagnosis value of Mtb‐specific perforin in pediatric patients with tuberculosis.

**Methods:**

Diagnostic performance of perforin levels induced by 6‐kDa early secreted antigen target (ESAT6) or culture filtered protein 10 (CFP10) were evaluated in eighty‐six samples from children participants by receiver operating characteristic curve analysis. Flow cytometry was used to detect the expression of perforin and INF‐γ of CD4^+^, CD8^+^ T cells in response to CFP10 stimulation.

**Results:**

After ex vivo stimulation, levels of ESAT6/CFP10‐specific perforin in LTBI patients were significantly higher than active TB (ATB) patients, non‐tuberculosis infection (non‐TB), and health control (HC) individuals. The diagnostic efficacy of CFP10‐specific perforin for TB diagnosis was significantly higher than ESAT6‐specific perforin and T‐SPOT assay, and when 0.74 ng/mL was taken as the cutoff value, the sensitivity, specificity, and accuracy were 97.83%, 87.5%, and 93.02%. CFP10‐specific perforin in both CD4^+^ and CD8^+^ T cells were significantly higher in ATB patients compared to HCs and further increased in LTBI patients. However, INF‐γ was mainly secreted by CD4^+^ T cells and showed no significant difference between LTBI and ATB patients. In addition, CFP10‐specific perforin can effectively distinguish between ATB and LTBI with the cutoff value of 1.80 ng/mL. Sensitivity and specificity were 88.46% and 85.62%, respectively.

**Conclusions:**

CFP10‐specific perforin may be used as a novel cellular immunity‐based diagnostic marker of pediatric patients with tuberculosis, and with the potential for discriminating ATB from LTBI.

## Graphical abstract

Diagnostic efficacy of CFP10‐specific perforin for TB diagnosis was higher than T‐SPOT assay. CFP10‐specific perforin effectively distinguishes between ATB and LTBI. CFP10‐specific perforin can be secreted by CD4^+^ and CD8^+^ T cells.

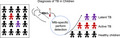

## 1 INTRODUCTION

Tuberculosis (TB) is one of the top 10 causes of death, and the leading cause from a single infectious pathogen (*Mycobacterium tuberculosis*, Mtb), ranking above HIV/AIDS. Pediatric patients account for an increasing proportion of TB cases in both high‐resource and low‐resource countries, with an estimated 1.1 million new cases in 2018.^1^ It is estimated that most active TB (ATB) cases originate from an initial latent TB infection (LTBI), a state of persistent immune response to stimulation by Mtb antigens without evidence of clinical manifestations of ATB.^2^ In contrast to adults, pediatric patients with LTBI are more likely to progress to ATB within the first year of primary infection^3^ and the efficacy of currently available treatments ranges from 60% to 90%.^2^ Thus, accurate and early identification of LTBI has become one of the key strategies to reduce TB incidence.^4^, ^5^

Clinical manifestations of TB, especially in infancy, are often non‐specific and similar with many common childhood diseases. Microbiological diagnosis of TB in pediatric patients remains a challenge as sputum is difficult to obtain and usually pauci‐bacillary, which resulted a poor sensitivity (<15%) of sputum smear microscopy in pediatric TB, even with optimized methods such as centrifugation of samples and use of fluorescent microscopy.^6^ While culture of Mtb is more sensitive than smear microscopy, bacteriological confirmation of pediatric TB is still less than 40%.^7^, ^8^ Automated real‐time nucleic acid amplification tests (Xpert MTB/RIF) are only positive in a proportion of children who have been clinically diagnosed with tuberculosis.^9^, ^10^, ^11^ Due to the difficulty in collecting satisfactory sputum samples from children, pediatric research has focused on finding new biomarkers in non‐sputum‐based samples for the detection of Mtb infection, including tuberculin skin test (TST) and interferon‐gamma release assays (IGRAs).^12^ Meta‐analyses revealed that both TST and IGRAs had higher specificities (84%‐98%) but low sensitivities (50%‐80%) in pediatric patients, and were unable to distinguish between ATB and LTBI.^13^, ^14^, ^15^ Importantly, TST are typically unresponsive during the first several weeks after infection, especially in infants and in cases of very severe or miliary ATB.^13^ Therefore, there is an urgent need to explore experimental methods for rapid diagnosis of tuberculosis in pediatric patients, especially for the differentiation of two different clinical status, LTBI and ATB.

Many studies have tried to use the combinations of different immunodiagnostic biomarkers such as EGF and TGF‐α; IL‐1RA, IP‐10, and VEGF; IL‐2 and INF‐γ; IL‐15 and MCP‐1; TNF‐α, IL‐12p40, and IL‐17; EGF, MIP‐1β, sCD40L, and IL‐1α to improve the sensitivity of TB diagnosis.^16^, ^17^ In addition, Comella‐del‐Barrio et al^18^ recently reported that the combination of INF‐γ, IP‐10, ferritin, and 25‐hydroxyvitamin D may have the potentiality to discriminate ATB from LTBI in pediatric patients. Although the diagnostic accuracy of these methods still need to be confirmed in multicenter studies, it is undeniable that soluble factors of PBMCs induced by Mtb‐specific antigen in vitro may be new markers with the potential for be used as new biomarkers for effectively diagnosis of pediatric patients with TB.

Host mainly depends on cellular immunity that mediated by CD4^+^ and CD8^+^ T lymphocytes to defense against Mtb infection. The cytotoxic molecules secreted by CD8^+^ T cells, such as granzyme and perforin, are directly involved in immune defense against Mtb^19^ and usually suppressed in granulomas within the tuberculosis lesions.^20^ In a multiple cohort to identify stage‐specific host responses to Mtb infection, Roshni et al^21^ reported that perforin‐expressing cells were significantly higher in LTBI than in normal and ATB patients, and were associated with the progression of tuberculosis. It is worthy to note that, unlike IFN‐γ mainly secreted by CD4^+^ T cells, perforin is not only secreted by CD8^+^ T cells, but also by CD4^+^ T cells. Laetitia et al^22^ reported that perforin‐expressing CD4^+^ T cells induced by Mtb‐specific antigens (Heparin‐binding hemagglutinin, HBHA) were found in most LTBI subjects and significantly higher than which in ATB patients. These results suggest that perforin may be a novel immune‐responsive biomarker for diagnosis of Mtb infection and the differentiation of LTBI and ATB. Therefore, we compared perforin levels induced by Mtb‐specific antigens in PBMCs of pediatric patients with TB in this study, and further evaluated their diagnostic potential for the differentiation of ATB and LTBI.

## 2 MATERIALS AND METHODS

### 2.1 Study design and patients

This study was carried out from January 12 to August 17, 2019, in Wuhan Children's Hospital and Wuhan Jinyintan Hospital (a hospital specializing in infectious diseases, Wuhan, China). Eighty‐six children (≤14 years old) were classified into the following four groups: healthy controls (HC); non‐TB controls; LTBI; and ATB, including pulmonary tuberculosis (PTB) and extrapulmonary tuberculosis (EPTB). The main characteristics of participants were shown in Table 1. Participants who had negative T‐SPOT.TB results and without any pulmonary symptoms or active disease were recruited as HC. Pediatric patients with clinical symptoms (fever and cough), bacterial or viral infection indicated by pathogen detection, and improved after anti‐infection treatment, but without evidence of ATB were recruited as the non‐TB controls. Non‐TB controls included 20 children with bronchopneumonia, including 13 cases with bacterial infection, 5 cases with *mycoplasma* infection, and 1 case with respiratory syncytial virus infection. Individuals with positive T‐SPOT.TB results but without clinical or radiographic evidence of ATB were diagnosed as LTBI.^23^ The diagnostic criteria of ATB include typical clinical symptoms (fever, cough, and weighting loss), laboratory isolation of Mtb in mycobacterial culture from sputum, broncho‐alveolar lavage fluid or plerual effusion, and/or acid‐fast staining, and/or Xpert MTB/RIF. The diagnosis of the 26 pediatric patients with ATB was given by a clinician after validation of these criteria associated with clinical symptoms. Participants with HIV or with solid organ transplantation or rheumatologic disease and receiving immunosuppressive treatment were excluded in the present study. This study was approved by the ethical committee of Wuhan Children's Hospital (WHCH 2019016). Written consents were obtained from all participants or their guardians.

**Table 1:** Clinical characteristics of the study participants

	TB		Control	
	ATB (n = 26)	LTBI (n = 20)	Non‐TB (n = 20)	HC (n = 20)
^a^Male (n, %)	12 (46.15%)	14 (70%)	12 (60%)	8 (40%)
^b^Age, median (IQR)	6.5 (2, 11.25)	6 (4, 11)	4 (3, 5)	6 (3, 9)
Under 5 y (n, %)	12 (46.15%)	9 (45%)	12 (65%)	9 (45%)
T‐SPOT positive (n, %)	11 (42.31%)	20 (100%)	0 (0%)	0 (%)
Infection site (n, %)				
Lung	19 (73.08%)			
Pleura	1 (3.85%)			
Bone	1 (3.85%)			
Lymph	2 (7.39%)			
Other	3 (11.54%)			

Abbreviation: IQR, Inter‐quartile range.

^a^ There are no significant differences in terms of sex among the groups (χ^2^ = 4.557, *P* = .275).

^b^ There are no significant differences in terms of age among the groups (*P* = .082).

### 2.2 PBMCs isolation and stimulation

2‐4 mL venous blood of all participants was collected into heparin lithium‐anticoagulant tubes, and PBMCs were separated by Ficoll‐Hypaque density gradient centrifugation. Then, 100 μL of fresh PBMCs (2.5 × 10^5^/well) was seeded in 96‐well plates (BD company) and were stimulated with 50 μL of ESAT6, CFP10, or AIM‐V medium (ESAT6 and CFP10 reagents were the same as TSPOT.TB kit, provided by Shanghai Fosun Medical Technology Co., Ltd.). The plates were incubated at 37°C for 16‐20 hours. After incubation, the supernatant was collected after centrifugation at 1301 *g*, and stored at −80°C for use.

### 2.3 T‐SPOT assay

The T‐SPOT assay was done according to the manufacture's clinical protocol (Shanghai Fosun Medical Technology Co., Ltd.).

### 2.4 ELISA for determination of perforin

Concentrations of perforin in culture supernatant were measured by a standard sandwich cytokine ELISA procedure according to the instruction of Human Perforin Precoated ELISA kit (Cag. # 1118302, Beijing Dakewe Biotechnology Co., Ltd.). Firstly, 100 μL/well of fivefold diluted culture supernatant or standards was added into ELISA plate and incubated for 2 hours at room temperature (18‐25°C). Then, 100 μL/well diluted biotinylated antibody (1:100) was added after 3 times washing and incubated for 1 hour at room temperature. Next, 100 μL/well of diluted Streptavidin‐HRP antibody (1:100) was added after 3 times washing and incubated for 30 minutes at room temperature. Lastly, the absorbance was measured by a microplate reader (Molecular Devices) at OD_450_ nm following TMB stain. The standard curve was drawn according to the standard samples; then, the concentration of perforin in the culture supernatant was calculated. Mtb‐specific perforin was defined by the following formula: Mtb‐specific perforin = perforin_ESAT6 or CFP10 stimulation_ − perforin_background_. Perforin_background_ represented the perforin levels in medium‐stimulated PBMCs.

### 2.5 Flow cytometry analysis

PBMCs (5 × 10^6^/well) were stimulated with CFP10 in AIM‐V medium in 6‐well flat bottom tissue culture plate at 37°C for 24 hours. After stimulation, the cells were stained for surface markers (PerCP‐anti‐CD45, FITC‐anti‐CD3, APC‐anti‐CD4, PE‐anti‐CD8; BD clinical Multi‐test™ IMK Kit) at room temperature for 15 minutes in the dark. Intracellular staining (BV‐510‐anti‐IFN‐γ, BV‐421‐anti‐perforin, BD Biosciences) was performed for 20 minutes at room temperature after fixation and permeabilization according to the manufacturer's instructions. Cells were finally resuspended in 500 μL PBS and acquired using a FACSCanto II (Becton, Dickinson and Company). At least 50 000 events were acquired per sample. Data analysis was performed using FlowJo version 7.6 software.

### 2.6 Statistical analysis

Data were analyzed using GraphPad Prism 6.0 (GraphPad) and MedCalc V19.1. Statistical significance was determined by the Mann‐Whitney *U* test. Proportions for categorical variables were compared using the chi‐square test, although the Fisher exact test was used when the data were limited. Receiver operating characteristic (ROC) analysis was performed to determine the best cutoff levels of Mtb‐specific perforin in the discrimination between ATB and LTBI. Area under the curve (AUC), sensitivity, and specificity were reported, as well as the 95% confidence intervals (CI). The optimal cutoff values were chosen as when Youden's index (YI = sensitivity +specificity − 1) was maximum. Statistical significance was determined as *P* < .05.

## 3 RESULTS

### 3.1 Levels of total perforin in PBMCs stimulated by ESAT6/CFP10

**Figure 1:**
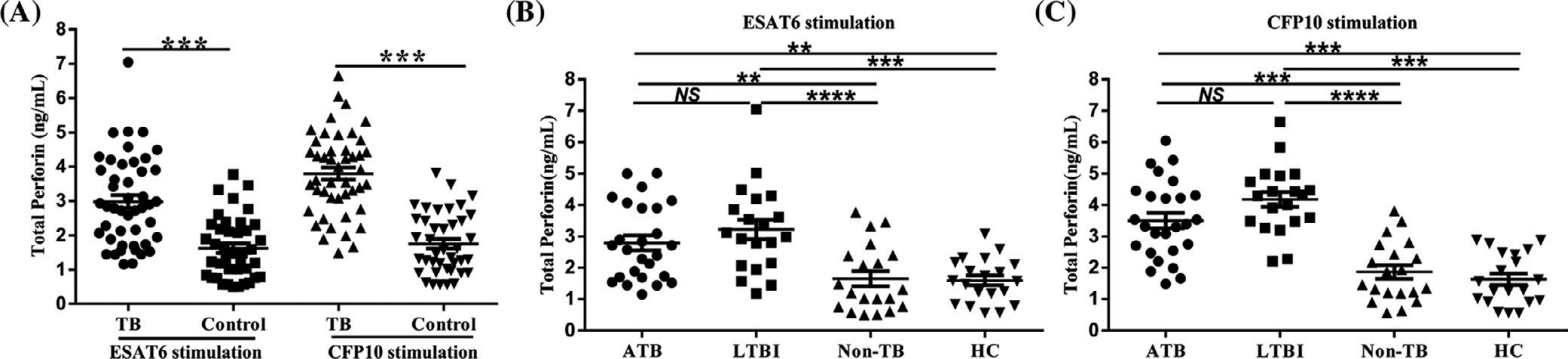
Levels of total perforin in PBMCs stimulated by ESAT6/CFP10. PBMCs obtained from ATB patients (n = 26), LTBI individuals (n = 20), healthy control subjects (n = 20), and non‐TB patients (n = 20) were stimulated with ESAT6 or CFP10 for 24 h. Levels of total perforin in supernatant were detected by ELISA. A, Combine ATB and LTBI as TB group, non‐TB, and HC as control group. Levels of total perforin in supernatant of ESAT6 or CFP10‐stimulated PBMCs isolated from TB group and control group. B, Levels of total perforin induced by ESAT6 in supernatant of PBMCs isolated from each indicated subgroup. C, Levels of total perforin induced by CFP10 in supernatant of PBMCs isolated from each indicated subgroup. Median values for each group of participants are represented by a horizontal bar. Statistical analysis was performed by the Mann‐Whitney *U* test. **P < .01, ***P < .001

To evaluate the potential of perforin for tuberculosis diagnosis, we firstly combined ATB and LTBI as TB group, non‐TB, and HC as control group. After stimulation with ESAT6 or CFP10, levels of total perforin were significantly increased in PBMCs of TB group (ESAT6: 2.98 ± 1.29 ng/mL, CFP10: 3.80 ± 1.21 ng/mL) compared with control group (ESAT6: 1.63 ± 0.89 ng/mL, CFP 10:1.75 ± 0.89 ng/mL), (*P* < .001), (Figure 1A). In subgroups analysis, the levels of total perforin were significantly increased in PBMCs of ATB patients (2.79 ± 1.20 ng/mL) and LTBI patients (3.22 ± 1.40 ng/mL) compared to healthy volunteer subjects (1.60 ± 0.71 ng/mL) and non‐TB patients (1.66 ± 1.06 ng/mL) after stimulation with ESAT6 (Figure 1B). However, total perforin secretion had no statistical difference between ATB patients and LTBI individuals (*P* = .268). Total perforin levels were also significantly increased in PBMCs of ATB patients (3.50 ± 1.24 ng/mL) and LTBI patients (4.18 ± 1.07 ng/mL) in response to CFP10 stimulation as compared to healthy control subjects (1.64 ± 0.82 ng/mL) and non‐TB patients (1.87 ± 0.99 ng/mL), and ATB patients still had no statistical difference as compared with LTBI individuals (*P* = .057), (Figure 1C). These data suggest that although levels of total perforin induced by ESAT6 or CFP10 in PBMCs were significantly increased in TB patients, total perforin levels may not have the potential to distinguish ATB from LTBI.

### 3.2 Levels of Mtb‐specific perforin in PBMCs stimulated by ESAT6/CFP10

**Figure 2:**
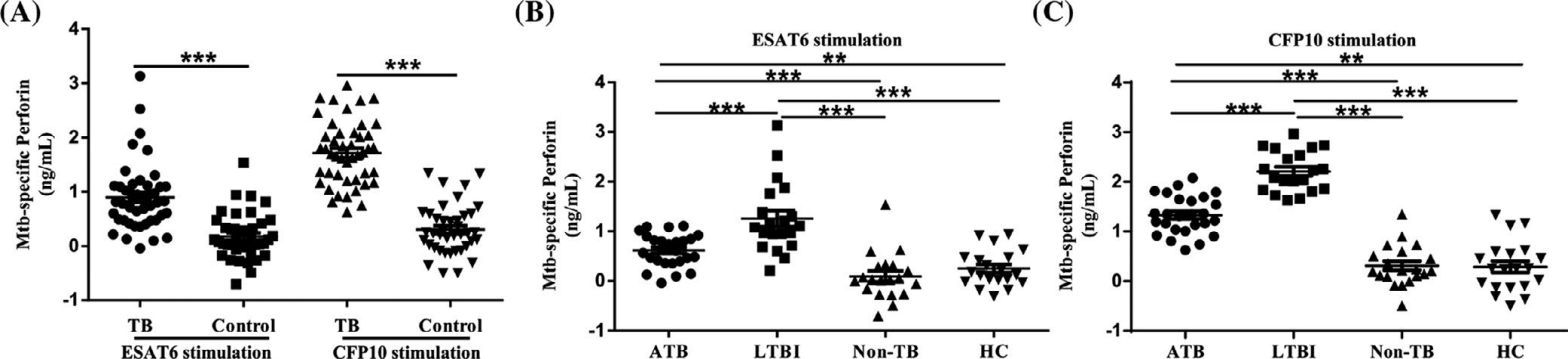
Levels of Mtb‐specific perforin in PBMCs stimulated by ESAT6/CFP10. Mtb‐specific perforin was calculated by subtracting background. PBMCs stimulated with medium alone were used as a background control. A, Levels of Mtb‐specific perforin in supernatant of ESAT6 or CFP10‐stimulated PBMCs isolated from TB group and control group. B, Levels of Mtb‐specific perforin induced by ESAT6 in supernatant of PBMCs isolated from each indicated subgroup. C, Levels of Mtb‐specific perforin induced by CFP10 in supernatant of PBMCs isolated from each indicated subgroup. Median values for each group of participants are represented by a horizontal bar. Statistical analysis was performed by the Mann‐Whitney *U* test. **P < .01, ***P < .001

Then, we further evaluated the difference of Mtb‐specific perforin levels among different groups. After subtracting the background, levels of ESAT6/CFP10‐specific perforin in TB group (ESAT6: 0.90 ± 0.61 ng/mL, CFP10: 1.71 ± 0.59 ng/mL) were significantly higher than that of control group (ESAT6: 0.17 ± 0.42 ng/mL, CFP10: 0.30 ± 0.46 ng/mL), (*P* < .001), (Figure 2A). In subgroups analysis, levels of ESAT6‐specific perforin were significantly increased in PBMCs of LTBI individuals (1.26 ± 0.71 ng/mL) compared to ATB patients (0.67 ± 0.39 ng/mL), healthy controls (0.25 ± 0.36 ng/mL), or non‐TB control subjects (0.09 ± 0.47 ng/mL), (*P* < .001), (Figure 2B). Similarly, CFP10‐specific perforin were increased in PBMCs of LTBI individuals (2.21 ± 0.41 ng/mL) compared to ATB patients (1.33 ± 0.38 ng/mL), healthy controls (0.29 ± 0.51 ng/mL), or non‐TB control subjects (0.31 ± 0.41 ng/mL), (*P* < .001), (Figure 2C). These data suggest that ESAT6/CFP10‐specific perforin in PBMCs not only distinguish TB from non‐TB/HC, but may also with the potential to discriminate between ATB and LTBI.

### 3.3 Comparison the diagnostic performance of total and Mtb‐specific perforin for TB diagnosis

**Figure 3:**
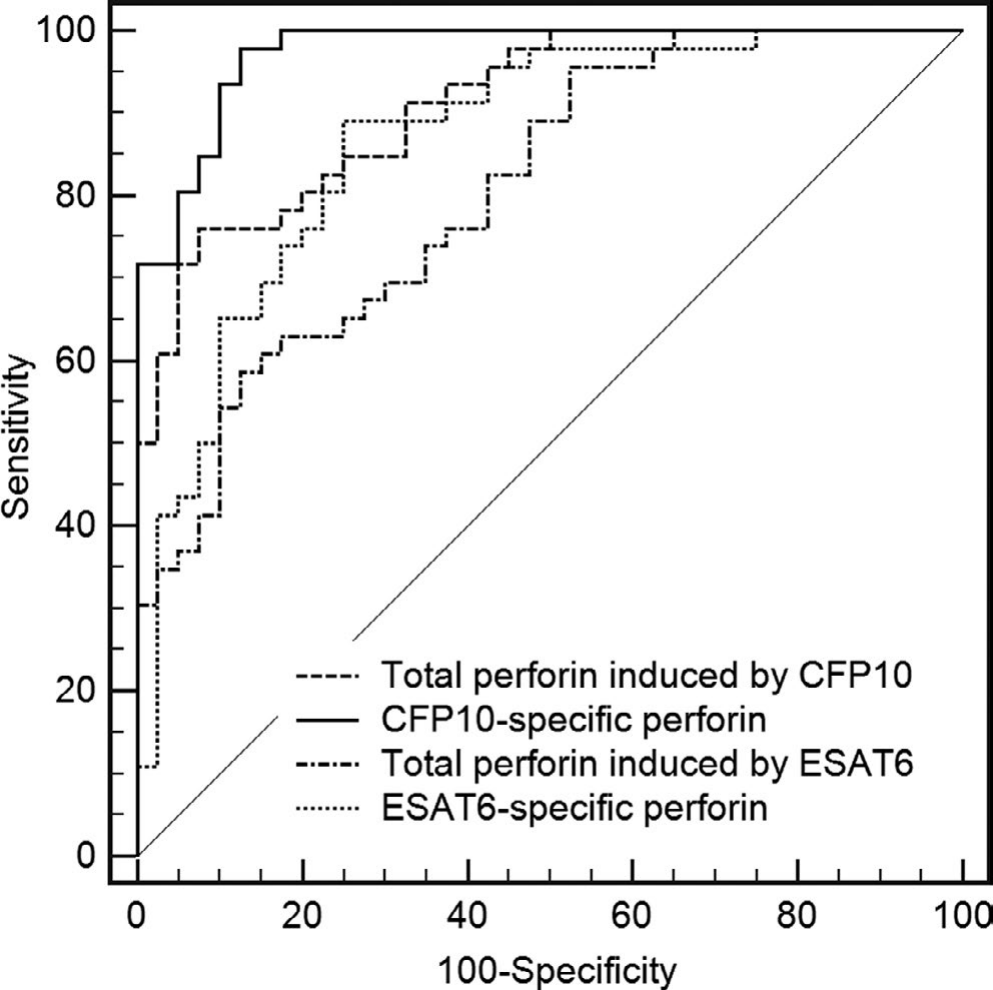
The receiver operating characteristic (ROC) curves (plotting sensitivity versus 1‐specificity) of total/Mtb‐specific perforin to discriminate patients with TB from control. The ROC analysis was performed for total/Mtb‐specific perforin to determine cutoff levels for the diagnosis of TB disease

Receiver operating characteristic analysis was then performed to determine the optimal cutoff values of total/Mtb‐specific perforin for the diagnosis of Mtb infection (Figure 3). When the cutoff value of total perforin induced by ESAT6 was set as 2.67 ng/mL, the sensitivity and specificity to discriminate TB patients (composed by ATB and LTBI groups) were 58.70% and 87.50%, respectively, and AUC was 0.81 (95% CI: 0.72‐0.90). With regard to CFP10 stimulation, ROC analysis demonstrated the AUC was 0.91 (95% CI: 0.85‐0.97), with sensitivity of 76.09% and specificity of 92.50% in diagnosing TB disease when the cutoff value was 2.99 ng/mL. The sensitivity and specificity of ESAT6‐specific perforin (cutoff value: 0.35 ng/mL) were 89.13% and 75.00%, and AUC was 0.87 (95% CI: 0.79‐0.94). The sensitivity and specificity of CFP10‐specific perforin (cutoff value: 0.74 ng/mL) were 97.83% and 87.50%, and AUC was 0.97 (95% CI: 0.95‐1.00). Differences between AUCs were analyzed using DeLong test. The AUC of CFP10‐specific perforins was significantly higher than that of total perforin induced by CFP10 and ESAT6, as well as ESAT6‐specific perforin (CFP10‐specific perforin vs Total perforin induced by CFP10, *P* = .019; CFP10‐specific perforin vs ESAT6‐specific perforin, *P* = .041; CFP10‐specific perforin vs Total perforin induced by ESAT6, *P* = .0004). These data suggest that CFP10‐specific perforin is a potential biomarker for the diagnosis of Mtb infection.

### 3.4 Comparison of the diagnostic performance of T‐SPOT and CFP10‐specific perforin

**Figure 4:**
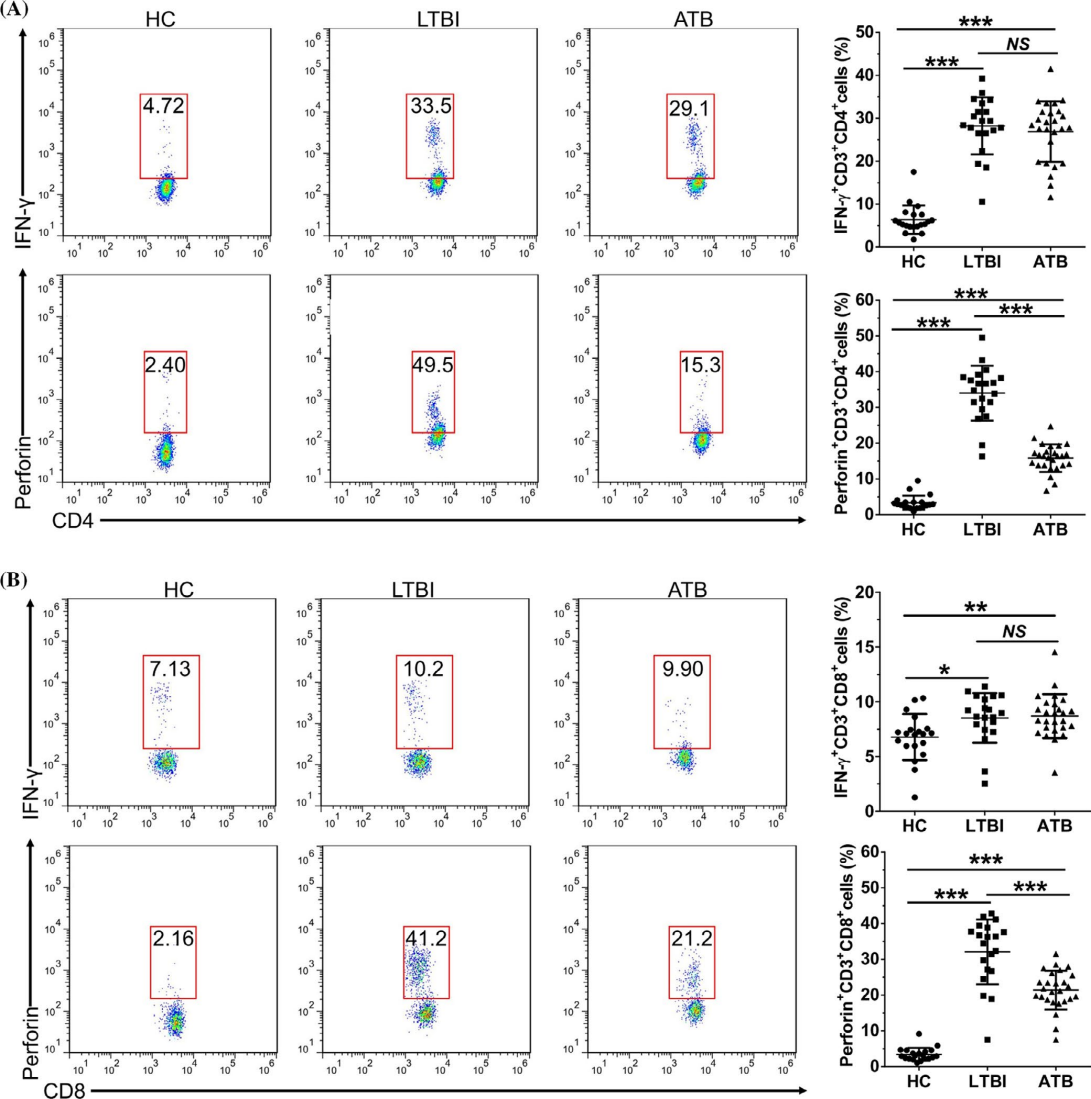
Qualitative analysis of perforin and INF‐γ expression ex vivo in CD4^+^/CD8^+^ T cells from patients with ATB, LTBI, and HC individuals. Representative FACS plot of INF‐γ and perforin expression ex vivo in CD4^+^ T (A) and CD8^+^ T cells (B) after stimulation with CFP10. Each dot corresponds to the result from an individual child, and horizontal bars represent the medians of the results. *P < .05, **P < .01, ***P < .001

Next, we further compared the difference of diagnostic performance between CFP10‐specific perforin and clinical T‐SPOT.TB (to detect Mtb infection regardless of active or latent status). As shown in Table 2, when 0.74 ng/mL was used as the optimal cutoff value, the sensitivity, specificity, negative predictive value (NPV), and accuracy of CFP10‐specific perforin were higher than those of T‐SPOT, while there was no significant difference in the positive predictive value (PPV) (*P* = .069). To clarify the potential reason, we further analyzed perforin and INF‐γ expression ex vivo in CD4^+^/CD8^+^ T cells from patients with ATB, LTBI, and HC individuals. Perforin in CD4^+^ T cells (34.03% ± 7.70%, 15.84% ± 3.87%, Figure 4A) and CD8^+^ T cells (32.09% ± 9.08%, 21.40% ± 5.44%, Figure 4B) from LTBI and ATB patients in response to CFP10 stimulation were significantly higher than which in HC individuals (3.37% ± 2.01%, 3.44% ± 1.80%), (*P* < .001). Importantly, perforin induced in CD4^+^ T cells (15.84% ± 3.87% vs 34.03% ± 7.70%) and CD8^+^ T cells (21.40% ± 5.44% vs 32.09% ± 9.08%) from ATB patients were significantly lower than that from LTBI individuals. INF‐γ levels significantly increased in CD4^+^ T cells (28.24% ± 6.65% and 26.90% ± 7.04%, Figure 4A) of ATB and LTBI patients compared to HC (6.40% ± 3.35%), but only showed slight increase in CD8^+^ T cells (8.52% ± 2.27% vs 6.77% ± 2.10%, 9.10% ± 2.10% vs 6.77% ± 2.10%, Figure 4B). Furthermore, there was no significant difference in IFN‐γ expression between the ATB and LTBI groups (26.90% ± 7.04% vs 28.24% ± 6.65%, *P* = .825; 9.10% ± 2.10% vs 8.52% ± 2.27%, *P* = .549).

**Table 2:** Comparison between CFP10‐specific perforin and clinical T‐SPOT. TB for TB diagnosis

	T‐SPOT.TB	CFP10‐specific perforin	χ^2^	*P*
Sensitivity (%)	67.39 (31/46)	97.83 (45/46)	14.83	<.001
Specificity (%)	100.00 (40/40)	87.50 (35/40)	5.33	.021
PPV (%)	100.00 (31/31)	90.00 (45/50)	3.30	.069
NPV (%)	72.73 (40/55)	97.22 (35/36)	9.01	.003
Accuracy (%)	82.56 (71/86)	93.02 (80/86)	4.39	.036

Abbreviations: NPV, Negative predictive value; PPV, Positive predictive value.

### 3.5 Diagnostic performance of CFP10‐specific perforin for the discrimination of ATB and LTBI

**Figure 5:**
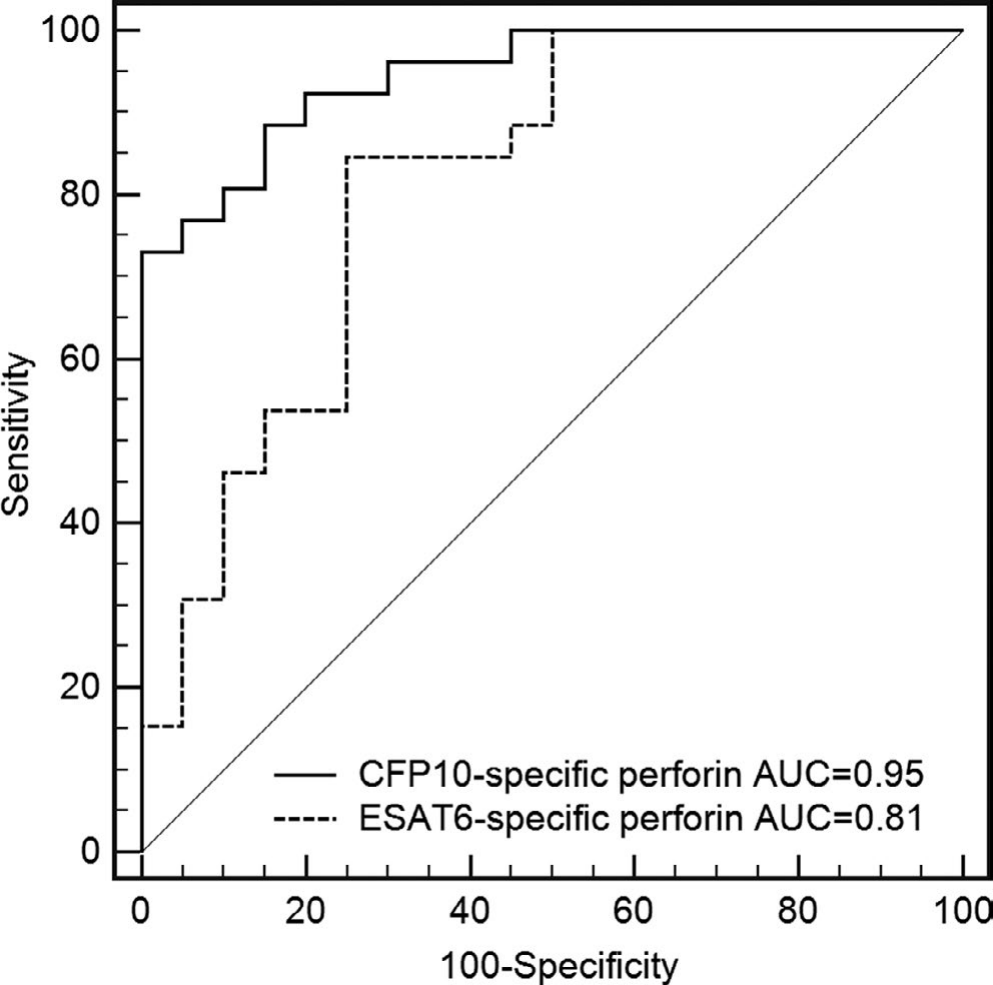
Receiver operating characteristic (ROC) for Mtb‐specific perforin as a classifier to distinguish between ATB and LTBI. The ROC analysis was performed for Mtb‐specific perforin to determine cutoff levels in distinguishing between ATB and LTBI

The AUC of ESAT6‐specific perforin was 0.81 (95% CI: 0.68‐0.94), with the sensitivity and specificity of 84.62% and 75.00%, when the cutoff value was 0.94 ng/mL (Figure 5), while the AUC of CFP10‐specific perforin was 0.95 (95% CI: 0.89‐1.00), with the sensitivity and specificity of 88.46% and 85.62%, when the cutoff value was 1.80 ng/mL. The AUC of CFP10‐specific perforin was slightly higher than ESAT6 as analyzed by Delong test (*P* = .0591).

## 4 DISCUSSION

IGRAs which measure ex vivo CD4^+^ T‐cell responses to Mtb‐specific antigens, including CFP10, EAST6, and TB7.7, have been widely used for detection of Mtb infection worldwide in recent years, including two main methods of T‐SPOT (Oxford Immunotec) and QFT‐GIT (QIAGEN). However, these assays have limited sensitivity in children, particularly in those under 5 years of age. In high‐income countries, the sensitivity of QFT‐GIT and T‐SPOT.TB is 79% and 67%, respectively, while in low‐income countries, the sensitivity is only 57% and 61%.^15^ In addition, Interferon‐gamma release assays cannot discriminate between LTBI and ATB.^24^ Previous studies have shown that multi‐cytokine (such as IL‐2, TNF‐α, and IL‐17) analysis in ex vivo stimulated PBMCs from LTBI and ATB patients exhibits potential to discriminate the two clinical states of Mtb infection.^25^ However, there is still no stable and reliable biomarker for clinical use to distinguish between ATB and LTBI for now.

Classically, perforin was reported to mainly exist in CTL and NK cells in the process of host defense against Mtb infection, which can lead to the dissolution of target cells and induce apoptosis of target cells. Rajeswari et al^26^ found that there was an increased percentage of perforin‐expressing CD8^+^ T cells in patients with TB, and Mtb strain H37Rv can promote the cytotoxic function of CD8^+^ T cells via inducing perforin release.^27^ In addition, Sousa et al^28^ reported that perforin knockout enhances the susceptibility of Mtb infection in mice. Laetitia et al^22^ further found that Mtb‐specific antigen HBHA induced perforin production of CD4^+^ T cells and which were able to differentiate LTBI subjects from patients with ATB. Moreover, QIAGEN recently developed an improved version of QFT‐GIT, QFT‐Plus, which is optimized with innovative tuberculosis‐specific antigens that elicit both CD8^+^ and CD4^+^ T‐cell responses (https://www.quantiferon.com/products/quantiferon-tb-gold-plus-qft-plus/), and has been confirmed to be more sensitive for detecting Mtb infection compared to QFT‐GIT.^29^ In our study, we found the sensitivity of Mtb‐specific perforin induced by ESAT6 or CFP10 were both significantly higher than that of IGRAs in previous meta‐analysis.^15^ In addition, the sensitivity, specificity, negative predictive value, and accuracy of CFP10‐specific perforin for the diagnosis of TB in pediatric patients were significantly better than that of T‐SPOT, which may be due to perforin can be secreted from both CD8^+^ T and CD4^+^ T cells.

To clarify the reason why diagnostic performance of CFP10‐specific perforin in pediatric patients were significantly better than T‐SPOT, we further compared the percentage of IFN‐γ or perforin producing CD4^+^ and CD8^+^ T cells. We found that perforin induced in both CD4^+^ T cells and CD8^+^ T cells from ATB patients was higher than that from healthy controls and further increased in LTBI individuals, while the expression of IFN‐γ was mainly expressed in CD4^+^ T cells and only showed a slight increase in CD8^+^ T cells from both ATB and LTBI patients compared to healthy controls. Myriam et al^30^ reported that IGRAs performed poorly for ATB diagnosis in children with advanced HIV infection (sensitivity was only 29%), which may be resulted by the fact that CD4‐response was impaired in HIV‐infected persons. Furthermore, considering lower capacity of CD4^+^ T cells to produce Mtb‐specific IFN‐γ, a lower cutoff level for defining QFT‐Plus‐positivity has been recommended in HIV‐positive pregnant women.^31^, ^32^ Thus, detection of ex vivo CFP10‐specific perforin may be a better method than IGRAs and which needs to be verified in multicenter in future.

In this study, total levels of perforin in response to ESAT6/CFP10 peptide stimulation in both ATB group and LTBI group were significantly higher than that of HC group and non‐TB group, but there was no significant difference between ATB and LTBI group, which may be caused by the release of perforin at different baseline levels in four groups. According to the methodology conducted by Wang Feng et al,^33^ they calculated Mtb‐specific TNF‐α by subtracting the background level of TNF‐α secreted by the unstimulated PBMCs, we found that diagnostic performance of Mtb‐specific perforin was substantially improved compared to total perforin for the diagnosis of TB disease and also discriminates between ATB and LTBI in this study. Interestingly, the diagnostic performance of CFP10‐specific perforin was significantly better than ESAT6, which may be due to the fact that ESAT6 protein can directly bind to TLR2 or beta‐2‐microglobulin (β‐2M) via the C‐terminal six amino acid residues, activate TBK1 and IRF3, thus inhibiting the antigen presentation of host immune cells and the release of inflammatory factors, while CFP10 has no immunosuppressive function.^34^, ^35^

Overall, the data presented in this study indicated that CFP10‐specific perforin is a novel cellular immunity‐based diagnostic marker of pediatric patients with tuberculosis, and it demonstrated high diagnostic potential in discriminating ATB and LTBI. The limitation of this study is that the small sample size comes from two hospitals, which may lead to sensitivity and specificity bias and our findings need to be further confirmed in a large, multicenter study of pediatric patients with tuberculosis.

## ETHICAL APPROVAL

This study was approved by the ethical committee of Wuhan Children's Hospital (WHCH 2019016).

## INFORMED CONSENT

Written consents were obtained from all participants or their guardians.
